# Mesoporous Silica Nanoparticles for the Treatment of Complex Bone Diseases: Bone Cancer, Bone Infection and Osteoporosis

**DOI:** 10.3390/pharmaceutics12010083

**Published:** 2020-01-20

**Authors:** Miguel Gisbert-Garzarán, Miguel Manzano, María Vallet-Regí

**Affiliations:** 1 Departamento de Química en Ciencias Farmacéuticas, Universidad Complutense de Madrid, Instituto de Investigación Sanitaria Hospital 12 de Octubre i + 12, Plaza Ramón y Cajal s/n, 28040 Madrid, Spain; mmanzano@ucm.es; 2 Networking Research Center on Bioengineering, Biomaterials and Nanomedicine (CIBER-BBN), 28029 Madrid, Spain

**Keywords:** mesoporous silica nanoparticles, mesoporous bioactive glasses, bone cancer, bone infection, bone regeneration, osteoporosis, stimuli-responsive drug delivery, targeted drug delivery

## Abstract

Bone diseases, such as bone cancer, bone infection and osteoporosis, constitute a major issue for modern societies as a consequence of their progressive ageing. Even though these pathologies can be currently treated in the clinic, some of those treatments present drawbacks that may lead to severe complications. For instance, chemotherapy lacks great tumor tissue selectivity, affecting healthy and diseased tissues. In addition, the inappropriate use of antimicrobials is leading to the appearance of drug-resistant bacteria and persistent biofilms, rendering current antibiotics useless. Furthermore, current antiosteoporotic treatments present many side effects as a consequence of their poor bioavailability and the need to use higher doses. In view of the existing evidence, the encapsulation and selective delivery to the diseased tissues of the different therapeutic compounds seem highly convenient. In this sense, silica-based mesoporous nanoparticles offer great loading capacity within their pores, the possibility of modifying the surface to target the particles to the malignant areas and great biocompatibility. This manuscript is intended to be a comprehensive review of the available literature on complex bone diseases treated with silica-based mesoporous nanoparticles—the further development of which and eventual translation into the clinic could bring significant benefits for our future society.

## 1. Introduction

In recent decades, nanotechnology has been applied to a variety of fields, ranging from novel electronic devices to the study of biological processes [1,2,3,4]. In particular, the application of nanotechnology to medicine, the so-called nanomedicine, has attracted the attention of many researchers, and it is expected to revolutionize the pharmaceutical and biotechnological fields in the near future [5,6,7].

The first developments in the field of nanomedicine were reported in the early 1960s, when liposomes were first proposed as carriers [8,9]. Since then, scientists have engineered many different nanocarriers to address effective delivery of therapeutics. Those nanoparticles can be classified as either organic or inorganic. Examples of organic nanocarriers include liposomes, which are amphiphilic lipids that rearrange in water to yield vesicles with an inner aqueous compartment surrounded by lipid bilayers [10]; polymeric nanoparticles produced from polymer chains showing different functionalities [11] or polymeric micelles composed by amphiphilic block copolymers able to rearrange in aqueous media [12]. Examples of inorganic nanocarriers include metal nanoparticles synthesized from noble metals, such as gold or silver [13]; carbon nanoparticles such as carbon nanotubes, fullerenes or mesoporous carbon nanoparticles [14] or silica-based mesoporous nanoparticles, which have been extensively studied owing to their capacity to load large amounts of therapeutic molecules [15]. The main advantages of silica-based mesoporous nanoparticles over other types of particles include the robustness of the silica framework, that allows the use of harsh reaction conditions for their modification, and their excellent textural properties. In fact, conventional polymeric nanoparticles usually present low drug capacity, usually less than 5% of total weight, whereas these silica-based mesoporous nanoparticles offer greater values [16,17]. The main disadvantage over other formulations would be the fact that the translation of these type of particles remains challenging. However, it should be mentioned that silica is “generally recognized as safe” by the US Food and Drug Administration (FDA), and it is often used as excipient in drug formulations and as dietary supplement [18,19]. In this sense, the administration of fenofibrate-loaded ordered mesoporous silica materials in men was found to be safe, and the doses were well tolerated by the patients [20]. In addition, small silica nanoparticles (c-dots, 7 nm) for imaging purposes were approved by the US FDA for a human clinical trial, demonstrating that they were well tolerated by the patients and accumulated in the tumor site [21]. In consequence, silica-based nanoparticles constitute a powerful and promising tool that might be promptly translated into the clinic.

This review will cover the application of silica-based mesoporous nanoparticles for the treatment of complex bone diseases, such as bone cancer, bone infection and osteoporosis. These pathologies are predominantly found in elderly people, who will constitute one-quarter of the European population by 2020 [22]. Then, bone diseases will definitely entail a significant impact on the health care systems and, consequently, bone-targeted nanomedicines, i.e., nanomedicines able to specifically reach bone diseases, could bring significant benefits for our future society.

## 2. Mesoporous Silica Materials

### 2.1. The Beginning of a New Era: Ordered Mesoporous Silica Materials

Ordered mesoporous silica materials were first reported in the early 1990s by Mobil Oil Corporation researchers [23] and scientists from Waseda university [24]. These bulk mesoporous materials have attracted great attention because they present (1) tunable and narrow pore size distributions (2–30 nm); (2) adjustable porous structures; (3) high specific surface areas (up to 1500 cm^2^/g); (4) high pore volumes (ca. 1 cm^3^/g); (5) high silanol density on the surface that allows further modifications [25,26]. Owing to their exquisite physico-chemical properties, mesoporous silica materials have been broadly applied in a number fields, including heavy metal adsorption [27,28], catalysis [29,30] or energy storage [31,32], among others.

In addition, these materials find broad application within the field of biomaterials, owing to their ability to adsorb molecules within their pores and release them in a sustained fashion. In fact, these materials have been widely studied since Prof. Vallet-Regí and coworkers first reported their suitability as drug delivery systems back in 2001 [33].

In light of their great properties and their potential biomedical application, researchers focused their efforts on translating those excellent features of bulk materials to the nanoscale dimension. As a result, mesoporous silica nanoparticles (MSNs) were developed soon after, opening the gates to multiple biomedical applications, such as controlled drug delivery [34,35], efficient gene transfection [36,37,38], antibacterial treatment [39,40] or bone tissue regeneration [41,42], among others.

### 2.2. Synthesis and Functionalization of Mesoporous Silica Nanoparticles

**Figure 1:**
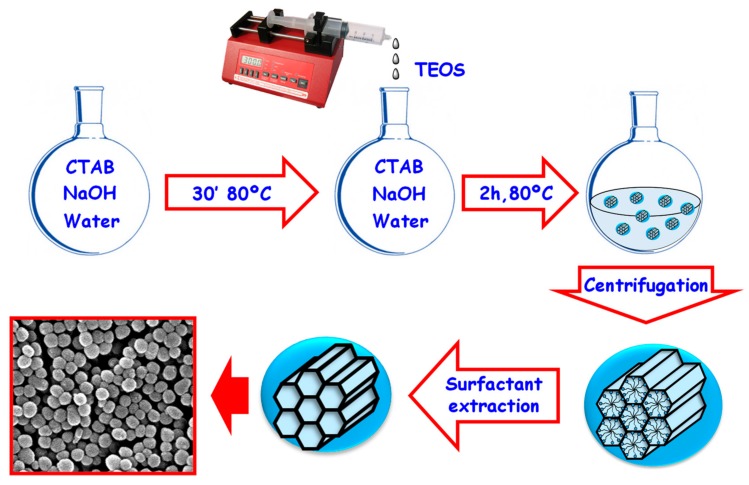
Synthesis of MCM-41 MSNs using a modification of the Stöber method. The surfactant molecules self-assemble forming rod-like micelles around which the silica precursors polymerize, leading to the formation of a silica backbone with hexagonally ordered mesopores. TEOS: Tetraethyl ortosilicate; CTAB: Cetyltrimethylammonium bromide.

The synthesis of MSNs is based on a modification of the Stöber method, which initially yielded micron-sized monodispersed and non-porous silica spheres [43]. In this sense, the addition of surfactants as structure-directing agents results in silica nanoparticles with excellent physico-chemical properties and showing porosity. This methodology allows obtaining homogenous nanoparticles within the range 50–300 nm [25]. The morphology and dimensions of these surfactant-templated mesoporous silicas can be tailored by controlling the reaction conditions (e.g., pH, temperature, surfactant concentration or silica precursor) [44]. As an example, a synthetic protocol for the synthesis of MCM-41 (Mobil Composition of Matter) MSNs is depicted in Figure 1.

The positively charged polar heads of the surfactant molecules interact with the negatively charged silica precursors, leading to the formation of the silica framework by means of the hydrolysis and condensation of the silica precursor onto the self-assembled rod-like surfactant micelles. Then, the organic template is removed using a solvent extraction method, yielding MSNs with empty pores ready to be filled with therapeutic molecules. This method is usually preferred over calcination, since the latter may cause irreversible aggregation of the particles and cytotoxic byproducts, limiting their potential application [45,46].

One of the most remarkable features of MSNs is their high density of silanol groups on the surface. These chemical groups allow the easy functionalization of the nanoparticles surface, usually using organosilanes bearing different functionalities (amine, carboxylic acid, thiol…), to increase the versatility of the produced nanocarriers. The particular organosilane employed allows tuning the interactions between the payload and the silica matrix, which might be beneficial for particular diseases [47,48]. The functionalization can be accomplished through two different approximations: post-synthesis or co-condensation. The post-synthesis method involves the modification of the surface after the synthesis. This approximation can lead to different groups inside and outside the pores, depending on whether the process is performed before or after removing the template. The co-condensation approach consists in the simultaneous addition of the silica precursor and the functional organosilane during the formation of the particles. This approximation can yield nanoparticles bearing various functional groups homogenously distributed throughout the silica backbone or biodegradable periodic nanoparticles with labile bonds within the silica framework [25].

### 2.3. Mesoporous Silica Nanoparticles as Smart Drug Delivery Systems

Aside from being biocompatible, any nanoparticle intended to be employed as a drug delivery system should fulfill some basic requirements, such as maximizing the amount of therapeutics loaded, minimizing premature release, reaching the target area and releasing the cargo on-demand only where needed. In this sense, the extraordinary textural properties of MSNs endow them with great loading capacities, being able to load huge amounts of therapeutic molecules within their pores, as demonstrated by Scanning Transmission Electron Microscopy [49]. In addition to serving as a drug reservoir, the silica matrix provides a protective shell for the molecules against potential pH- or enzymatic-mediated drug degradation in the organism.

**Figure 2:**
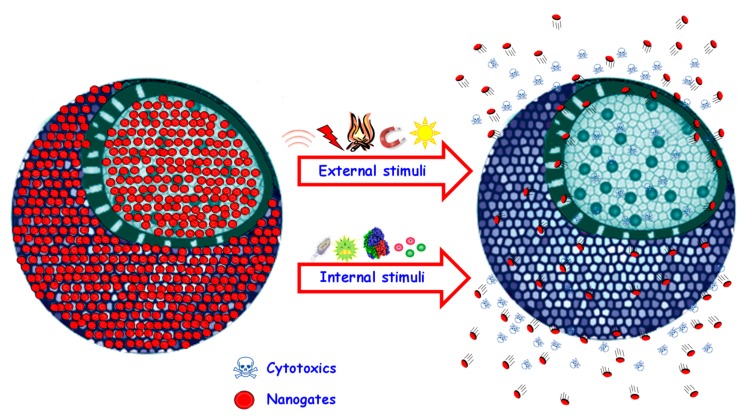
Schematic representation of stimuli-responsive MSNs. In response to the stimulus, the gatekeeper opens the pore entrances, triggering drug release. The origin of the stimulus can be internal (pH, enzymes, redox species, etc.) or external (magnetic fields, light, ultrasounds, etc.).

The loading of therapeutic molecules within MSNs can be easily accomplished as consequence of their open porous structure. However, this also means that the cargo molecules might easily diffuse out of the pores before reaching the target area. This premature release can be minimized using the so-called stimuli-responsive gatekeepers, which are structures able to open and close the pore entrances on-demand in response to certain stimuli [16,50,51,52]. In this manner, premature and non-specific drug release would be minimized, and the release would only take place upon application of a convenient stimulus at the diseased area (Figure 2).

Stimuli can be applied from inside or outside the organism. The use of internal stimuli is interesting because of the significant variations of various relevant biomarkers that can be found in some diseases. For instance, the pH of some subcellular organelles and that of the tumoral matrix are more acidic compared to the physiological value [53], and analogous behavior is observed in bacterial infections [54]. These pH variations have been employed to trigger the release from pH-responsive smart MSNs [55,56,57,58]. In addition, some enzymes, which have been observed to be overexpressed in osteoporotic [59] or tumoral scenarios [60,61], have the ability to cleave very specific peptidic sequences. In this sense, it is possible to use those peptides to close the pore entrances of MSNs and trigger drug release only in those situations, where the enzymes are overexpressed [62,63,64]. Another relevant example of an internal stimulus is the overexpression of redox species in the cytoplasm of tumoral cells compared to the extracellular fluids [65,66], which has been employed to initiate drug release from different redox-responsive MSNs [67,68,69].

External stimuli, which should be innocuous to the organism, have also attracted great attention. Their main advantage is that they would allow the application of the stimulus directly by the clinician, thereby providing a much higher control of the release kinetics. For instance, the generation of heat through the application of alternating magnetic fields has been employed trigger drug release from MSNs and generate hyperthermia-mediated cell death [70,71,72]. The use of light (ultraviolet, visible, near-infrared) has also attracted the attention of many researchers, and constitutes a non-invasive method to trigger the release from MSNs [73,74,75]. Another relevant example of a non-invasive and innocuous stimulus is ultrasounds, which have been successfully employed to externally trigger the payload release from MSNs [76,77,78].

### 2.4. Biodistribution and Biodegradation of Mesoporous Silica Nanoparticles

The most common routes of administration of the above-mentioned smart nanoparticles are intravenous, subcutaneous or localized injections in the target area. In particular, the intravenous administration leads to the rapid delivery and distribution of the particles throughout the organism, albeit it entails challenging issues. For instance, the particles administration leads to the formation of a protein corona around them that defines their biological entity. This protein coating might limit the functionality of the nanoparticles and enables their recognition by the organism, triggering their removal by the mononuclear phagocyte system and decreasing the efficiency of the treatments [79]. An effective approximation to overcome that issue would be the modification of the nanoparticles with hydrophilic polymers, such as poly(ethylene glycol) (PEG), which might help reduce the amount of proteins adsorbed onto the nanoparticles by creating a hydrophilic layer, enhancing their colloidal stability and increasing their circulating half-life [80,81,82]. In this sense, it has been shown in murine models that non-PEGylated MSNs rapidly accumulate in the lung, liver and spleen while their PEGylated counterparts show increased circulating half-life [83,84].

Besides the effect of PEGylation on the particles biodistribution, there are other relevant parameters that influence the final fate of MSNs. For instance, it has been shown in vivo that the larger the nanoparticles the faster their excretion [84]. In addition, it has been observed that, unlike spherical particles, those presenting elongated or cylindrical shapes undergo faster clearance from the bloodstream [85]. Finally, the surface charge is a key parameter since it determines the interaction of the particles with the surrounding media. In this sense, it has been shown that positively charged nanoparticles are more prone to undergo opsonization and subsequent clearance than their slightly negative or neutral counterparts [86,87].

Aside from achieving effective accumulation at the diseased area, it would be desirable that the MSNs degrade somehow to facilitate their excretion after exerting their therapeutic activity. In this sense, the dissolution rate of the silica backbone is a key factor for their elimination. Silica-based mesoporous nanoparticles are composed of polycondensed silica tetrahedrons (SiO_4_) interconnected by siloxane bonds (–Si–O–Si–) and presenting silanol groups (–Si–OH) on the surface. The silica dissolution is consequence of the nucleophilic attack of water to the siloxane and silanol groups, generating biocompatible silicic acid as by-product that can be excreted through the urine [88]. The dissolution rate depends on the particular characteristics of the particles and can be tuned through the introduction of organic modifications on the surface. Those modifications have been shown not to affect the biodistribution and biocompatibility of the MSNs [85].

## 3. Mesoporous Silica Nanoparticles for the Treatment of Bone Cancer

### 3.1. General Concepts on Bone Cancer and Bone Metastasis

Cancer is the term given to a group of diseases sharing an unstoppable cell division and with potential to spread in other organs and tissues. It is a leading cause of mortality worldwide and its prevalence is progressively increasing, with 1.7 million of estimated new cases and 600,000 cases of estimated deaths only in the United States in 2019 [89].

Bone-related tumors fall into primary bone tumors and metastatic bone tumors. They are considered to be highly deadly even though chemotherapy has improved the patient survival for sarcomas [90]. The most common malignant primary bone tumors are osteosarcoma, chondrosarcoma and Ewing sarcoma, which account for 70% of such malignancies. They originate in the bone, where mesenchymal stem cells behave both as ontogenic progenitor tumor cells and stromal cells that contribute to tumor development. The stroma of these tumors comprises osteoblasts, osteoclasts, endothelial and immune cells and mesenchymal stem cells. In particular, osteoclast have grabbed great attention because their activity (bone destruction) can be metabolically enhanced directly by tumor cells and, reversibly, the presence of osteoclasts boosts the aggressiveness of cancer cells [91].

Metastasis is the spread of cancer cells from a primary tumor to distant sites to create secondary tumors. It is a stage of the disease usually considered to be incurable with mainly palliative treatments [92]. Its origin is the pre-metastatic niche, which is an environment in a secondary organ induced by the primary cancer cells that provides favorable conditions for the growth of tumoral cells [93]. The exact mechanism of that metastatic organotropism remains unclear, but it is thought to be related with tumor-derived exosomes. Exosomes are nanometric membrane-bound vesicles secreted by tumors cells that contain functional biomolecules, such as proteins, RNA, DNA and lipids [94]. In this sense, it has been reported that tumor exosome integrins can determine organotropic metastasis by fusing with organ-specific resident cells to stablish the pre-metastatic niche. Once uptaken, they induce cellular changes in the target organ (through the activation of Scr phosphorylation and pro-inflammatory S100), thus promoting cancer cell colonization and organ-specific metastasis [95].

A characteristic feature of this disease is that some types of cancer cells preferentially migrate and induce metastasis to specific organs [94]. In this sense, breast and prostate tumors normally lead to bone metastases, which are secondary tumors formed when primary tumor cells home to the skeleton [96,97]. Cancer cells can leave the primary tumor site owing to the poor adhesion among each other in the tumoral matrix [98]. Once colonized the bone, tumor cells secrete proteins that interact with resident cells in the bone marrow to induce the differentiation, recruitment and activation of osteoblasts and osteoclasts. Then, during the bone resorption the calcium ions and the growth factors secreted from the mineralized bone matrix promote tumor cell growth, leading to vicious cycle that supports tumor growth in bone and subsequent fatal outcome [93].

It is believed that, when primary tumor cells migrate, the interaction of these disseminated cells with the new microenvironment determines whether they will proliferate to form a secondary tumor or undergo growth arrest and subsequent dormancy. Dormant cells are cells that stop dividing but still survive in a quiescent state, waiting for the appropriate environmental conditions to re-enter the cell cycle again [99]. These cells are clinically undetectable and, consequently, constitute a major issue for future tumor recurrence and metastases [100]. Current pharmacological approximations are aimed at maintaining cancer cells in the dormant state; reactivating dormant cells to increase their susceptibility to drugs; and eliminating cancer cells. Those strategies rely on the modulation of certain factors present on or secreted by the dormant cells in such a way that their overexpression of inhibition affects the fate of those dormant cells [101]. In this sense, the use of mesoporous silica nanoparticles might be interesting to enhance those treatments, as they could be employed to load therapeutic agents able to modulate the expression of those factors. In addition, they could be employed to co-load those agents with antitumoral drugs, consequently enhancing the efficacy of the treatments and minimizing tumor recurrence.

### 3.2. Nanotechnology for Cancer Treatment

Current anticancer treatments mainly rely on chemotherapy, radiotherapy and/or surgery [102,103,104]. Those treatments, yet effective in many cases, present several drawbacks. In particular, chemotherapy lacks great tumor tissue selectivity, leading to non-specific drug distribution and side effects. In this sense, nanoparticles have emerged as a powerful tool to encapsulate drugs and reduce side effects [105,106,107].

**Figure 3:**
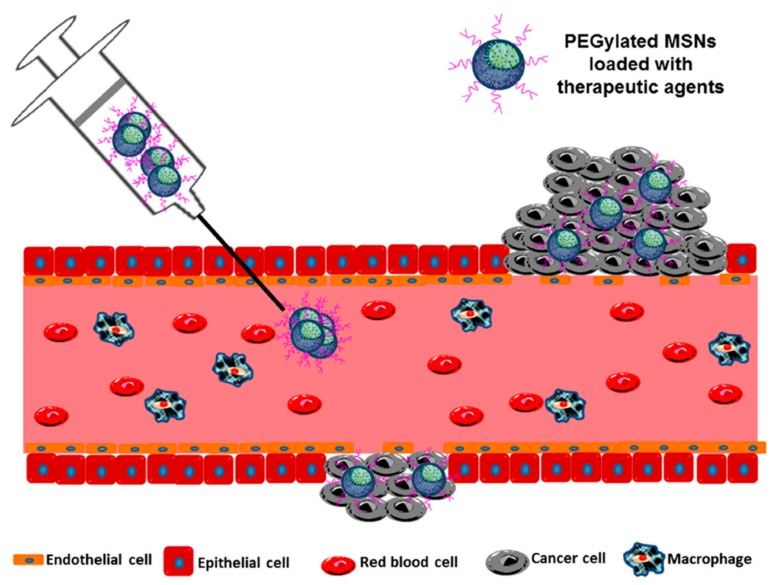
The Enhanced Permeability and Retention (EPR) effect. Nanoparticles passively accumulate in the tumor owing to the presence of fenestration in the tumor blood vessels. Once there, the particles remain in the tissue for long periods of time as a consequence of the poor lymphatic drainage. Reproduced from [110] with permission of MDPI, 2015.

The rationale behind the use of nanoparticles in cancer treatment relies on the Enhanced Permeability and Retention effect (EPR effect), which is the basis of some commercialized nanomedicines [108]. The EPR effect, first reported by Maeda and coworkers [109], promotes the passive accumulation of nanoparticles in solid tumors as a result of the hypervasculature, the enhanced permeability and the poor lymphatic drainage found in many tumors (Figure 3).

Owing to the uncontrolled angiogenesis, the newly formed vessels present an abnormal architecture, including wide fenestrations (200–2000 nm endothelial cell–cell gaps), irregular vascular alignment or lack of smooth muscle layer, among others. As a result, molecules larger than 40 kDa leak out from them and accumulate in the extravascular tumoral tissues. On the contrary, healthy tissues do not show this abnormal development and no accumulation is observed, thus creating a differential selectivity for cancer tissues [111]. In addition, unlike normal tissues where the extracellular fluid is constantly removed, tumors present defective lymphatic drainage and the accumulated macromolecules tend to remain in the tumoral mass for longer periods of time [112].

The magnitude of the EPR effect in humans highly depends on the particularities of the patient and the tumor [113] although some alternative strategies, such as tumor-homing peptides or some types of cells, are currently being explored to overcome the lack of EPR effect.

These alternative approximations have successfully been evaluated using in vivo tumor models, demonstrating the suitability of using MSNs for tumor drug delivery. In this sense, tumor-homing peptides (e.g., iRGD, iNGR) not only induce spontaneous accumulation of nanoparticles in the tumor tissues, but also enhance their diffusion into the tumoral mass [114,115]. In addition, there are certain types of cells with migratory properties that can transport nanoparticles directly to tumors tissues. For instance, nanoparticles can be attached to hypoxic bacteria that migrate to the hypoxic areas of tumors [116,117]. In addition, mesenchymal stem cells have been shown to migrate to tumors in response to the secretion of various signaling molecules. Then, a smart strategy is to induce the internalization of drug-loaded nanoparticles within these cells to then delivering them specifically to tumor tissues [118,119,120,121].

Besides delivering the nanoparticles to malignant tissues, the carriers can be engineered so that they preferentially recognize cancer cells over healthy cells. This targeting strategy relies on the overexpression of some receptors only on the membrane of tumoral cells. Examples of this approach include the functionalization of the particles with antibodies [122,123], proteins [69,124], small molecules [125,126,127,128,129] or peptides [77,130,131], among others.

### 3.3. Targeting Bone-Localized Tumors with Mesoporous Silica Nanoparticles

Addressing nanoparticles to bone metastases is challenging, as small metastases are poorly vascularized and, consequently, the magnitude of the EPR effect is low compared to big solid tumors [132]. A smart approximation would be the modification of the particles with targeting molecules with high affinity towards calcium phosphate surfaces (bone tissue), such as bisphosphonates [133], to complement the EPR effect. In this sense, the surface modification with the bisphosphonate zoledronate has been proved to be effective in delivering MSNs to bone metastases originated from lung [134] and breast cancer [135].

**Figure 4:**
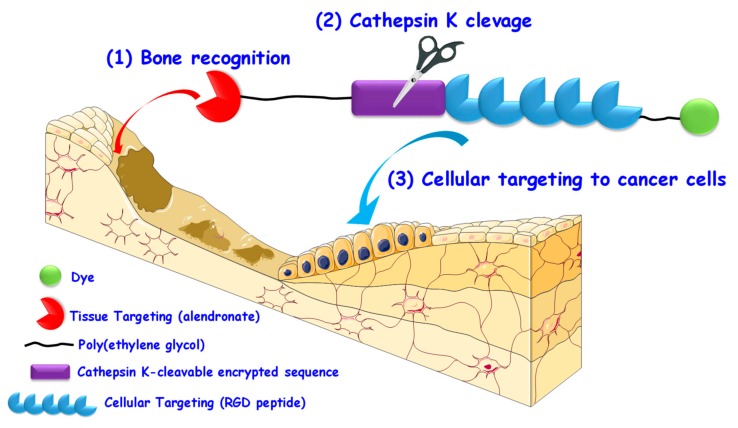
Encrypted approach for the sequential targeting of bone cancer tissue and cancer cells. (1) The presence of a bone targeting agent (alendronate) would help accumulate the nanomedicines in the bone tumor tissue; (2) Once there, the overexpressed cathepsin K would cleave a specific peptidic sequence, (3) exposing the RGD (arginine-glycine-aspartic) motif, which is able to promote the selective uptake of nanomedicine by sarcoma tumoral cells.

Besides targeting the particles to bone tissue, it would be desirable for the nanomedicines to be subsequently internalized only by the tumoral cells. In this sense, our group recently reported a smart approximation for the sequential targeting of bone tumors or bone metastases that could be easily implemented into any nanomedicine (Figure 4) [136].

As observed in Figure 4, the system is composed of two targeting agents and employs PEG chains to mimic a nanocarrier. The first one is the bisphosphonate alendronate, which can bind bone tissue. Then, there is a peptidic fragment containing a cathepsin K-cleavable sequence followed by the RGD motif, which is able to promote the selective internalization in osteosarcoma cells thanks to the overexpression of αβ integrins. In this manner, the alendronate molecule would help the EPR effect to accumulate the nanomedicines in the bone tumor tissue. Once there, cathepsin K, which is overexpressed in bone tumors and bone metastases, would cleave the encrypting sequence, thereby exposing the RGD motif and triggering the preferential uptake of the nanomedicines.

As it happens with many other cancer cells, bone tumoral cells overexpress specific receptors that can be targeted using conveniently engineered MSNs. Aside from targeting MSNs to osteosarcoma [137], the RGD motif can also be employed to recognize endothelial cells, which can help MSNs target the tumor endothelium of fibrosarcoma to then eliminate the cancerous cells using multimodal therapy [138]. In this sense, folic acid can be employed to target overexpressed folate receptors in fibrosarcoma [139] and osteosarcoma cells [126]. In addition, the modification of MSNs with a glucose analog enhances their accumulation in bone tumor cells, as a consequence of their great glucose consumption due to the high metabolic demand of tumors [140]. Some surface receptors, such as the CD11c, can also be targeted using specific antibodies, which are able to trigger the selective internalization of MSNs in osteosarcoma [141].

The decoration of MSNs with proteins can also increase their cellular uptake. For instance, the lectin concanavalin A binds overexpressed sialic acid residues to promote the cellular uptake of pH-responsive MSNs in osteosarcoma cells [142]. Transferrin receptors are overexpressed in fibrosarcoma cells and, consequently, the protein transferrin can be employed to enhance the uptake of MSNs in those bone tumoral cells [124].

Besides employing active targeting moieties, MSNs can be internalized via electrostatic interactions with the negatively charged cell membrane. The positively charged surface can be shielded using PEG, which can be detached using a cleavable bond. The charge is exposed again upon application of ultrasounds, which triggers the nanoparticles uptake after the accumulation in the solid bone tumor via EPR effect [143].

### 3.4. Controlled Release of Therapeutics in Bone Tumors with Mesoporous Silica Nanoparticles

There are various examples of the suitability of using silica-based mesoporous nanomatrices for the delivery of antitumoral [144,145,146,147,148] or imaging agents [137,149,150] to bone cancer cells. Moreover, researchers have taken advantage of the features of the bone tumoral environment to design stimuli-responsive MSNs for the treatment of sarcomas. Among the internal stimuli, the acidic environment of the lysosomes can be employed to trigger drug release from pH-responsive polymer-coated MSNs [142] or pulsatile on-off MSNs with pore entrances that are sealed with pH-responsive nanovalves [78]. In addition, it is possible to load immunotherapy agents within the pores of pH-responsive lipid-coated MSNs for synergistic chemo-immunotherapy [151]. In addition to pH variations, the enzyme alkaline phosphatase, which is characteristic bone-related tumors, can be employed to degrade the gatekeepers of silica-based mesoporous glasses [152]. Moreover, the esterase enzymes can also be employed to cleave the nanocaps of MSNs [126].

There are some examples of the use of light to trigger drug release from MSNs in bone tumor scenarios. For instance, ultraviolet light can be employed to cleave light-responsive bonds connected to transferrin, which acts as both gatekeeper and targeting agent, triggering drug release [124]. In addition, porphyrins can be engineered as gatekeepers using a linker cleavable in the presence of singlet oxygen, which are self-produced by the porphyrin caps upon application of visible light [73].

Aside from delivering small therapeutic molecules, MSNs allow the effective delivery of proteins [153] or DNA strands [154] into bone cancer cells. There is a type of nucleic acids, small interfering RNA (siRNA), that triggers the knockdown of specific and relevant proteins, which makes them useful for the treatment of various diseases [155]. Unfortunately, siRNAs have short half-life, poor penetration through cell membranes and easily degrade upon RNase action in the organism [156]. For that reason, the use of MSNs as protective shell for these nucleic acids have been widely explored. In this sense, the polo-like kinase 1, which is an essential gene for the correct execution of cell division [157], is overexpressed in bone tumors and has been targeted with great efficacy using siRNA-loaded MSNs [158,159,160,161].

A summary of all the nanocarriers described here for bone tumors is summarized in Table 1.

## 4. Mesoporous Silica Nanoparticles for the Treatment of Bone Infection

### 4.1. General Concepts on Bacterial Bone Infections

**Figure 5:**
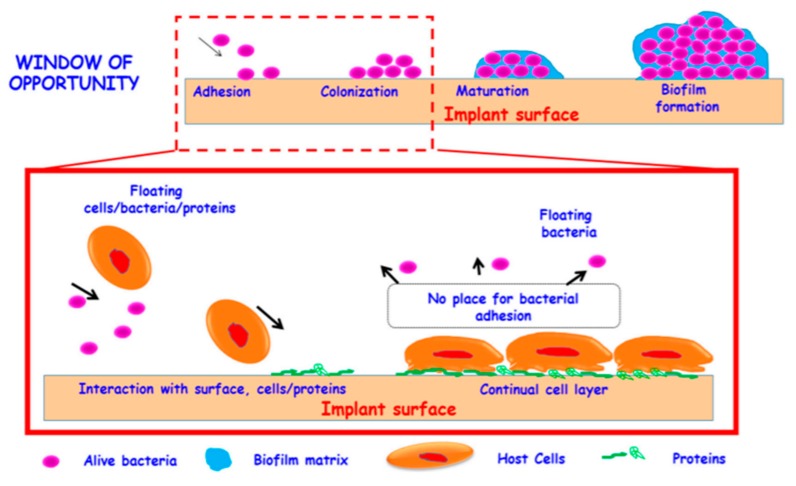
Schematic representation of biofilm formation on an implant surface. The process involves four steps: (1) bacterial adhesion, (2) bacterial growth, (3) maturation and (4) biofilm formation. In addition, bacteria may leak out from the matrix and lead to bacterial dispersion. The first stages constitute a window of opportunity, in which it is still possible to prevent biofilm formation. Reproduced from [40] with permission of MDPI, 2018.

Bone infection is a major issue for health care systems and entails important socioeconomic implications [162]. The appearance of bone infections is directly related with the progressive ageing of current society and, consequently, the increased use of implantable medical devices and their potential bacterial contamination. These infections are mainly caused by *Staphylococcus epidermis*, *Staphylococcus aureus*, *Escherichia coli* and *Pseudomonas aeruginosa* [40]. Regular bacteria can be relatively easy eliminated using antibiotics. However, the inappropriate use of those antimicrobials is progressively leading to more cases of drug-resistant bacteria, which are expected to cause more than 10 million deaths by 2050 [163]. This antimicrobial resistance induces uncontrolled bacterial growth and formation of persistent biofilms. Biofilms are communities of microorganisms embedded in a self-produced polysaccharide matrix [164]. This protective matrix endows them with resistance to antibiotics and host immune systems that, otherwise, would eliminate bacteria in their planktonic state (free-floating bacteria) [165]. The biofilm—related antimicrobial resistance relies, not only on the physical hindrance of the matrix, but also on (1) the presence of bacterial and host DNA and proteins that may increase the shielding capacity of the matrix [166]; (2) the presence of bacteria with different acquired resistances and antibiotic sensitivities [167]; (3) the development of efflux pumps [168]; (4) the presence of enzymes able to degrade antimicrobials [169]; (5) the establishment of quorum sensing (bacteria-bacteria communication) [170]. The process of biofilm formation is depicted in Figure 5.

The formation of the biofilm comprises 4 steps: (1) adhesion of bacteria to the implant surface; (2) bacterial growth in multiple bacterial layers; (3) maturation; (4) final biofilm formation. In addition, bacteria detach from the biofilm to then colonize other areas and induce further infections [171]. As observed in Figure 5, during the first phases of biofilm formation, the individual microorganisms are floating on the implant, reversibly interacting with the surface. In consequence, these stages constitute a window of opportunity that clinicians should take advantage of to prevent irreversible biofilm formation and subsequent resistance [40].

### 4.2. Preventing Protein and Bacterial Adhesion and Biofilm Formation: Zwitterionic Mesoporous Silica Nanoparticles

**Figure 6:**
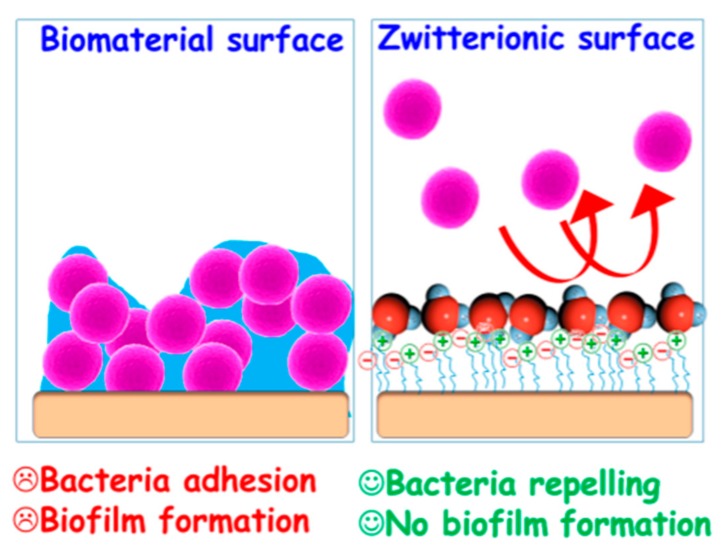
Schematic representation of bacterial colonization in standard surfaces vs. *zwitterionic* surfaces. Unlike in unmodified surfaces, *zwitterionic* materials create a hydration layer that prevents bacterial adhesion and biofilm formation. Reproduced from [40] with permission of MDPI, 2018.

In view of the existing evidence in the previous subsection, avoiding bacterial contamination of implants constitutes a major concern. In this sense, the development of the so-called *zwitterionic* materials has fueled the design of antifouling nanostructured materials able to prevent protein adsorption, bacterial adhesion and biofilm formation (Figure 6).

*Zwitterionic* surfaces are characterized by an equal number of negative and positive charges, so the net charge is expected to be neutral. This neutrality leads to the formation of a hydration layer onto the surface that physically hampers adhesion and biofilm formation [172]. In fact, owing to the reduced protein adsorption, *zwitterionic* functionalizations have also been postulated as substitutes for PEGylation [173], which might be beneficial to overcome the growing appearance of anti-PEG antibodies [174].

The first example of mesoporous silica materials with *zwitterionic* behavior was reported by our group back in 2010, using SBA-15 mesoporous materials modified with randomly distributed amino and carboxylic acid short chains on the surface that resulted in significantly lower protein adhesion [175]. A similar approach using amino and phosphonate groups was recently reported, yielding MSNs with extremely low protein adsorption and excellent antibacterial properties. In addition, the nanoparticles showed great biocompatibility with preosteoblasts, assuring their biocompatibility for the treatment of bone infection [176]. Interestingly, this *zwitterionic* approach using two small molecules can be employed to design pH-responsive gatekeepers by taking advantage of the interaction between both short chains, which interact at physiological pH and experience repulsion forces at acid pH [177].

Aside from merging molecules with opposite charges, there are molecules that are *zwitterionic* in nature. In this sense, the modification of MSNs with phosphorylcholine groups yields nanoparticles showing reduced protein adsorption and able to provide sustained drug release in response to changes in pH [178]. An analogous approximation is the modification of MSNs with sulfobetaine groups to prevent protein adhesion [179]. Moreover, it is possible to polymerize this kind of *zwitterionic* molecules to yield polymer-coated nanoparticles with low protein binding affinity [180]. In addition, there are some amino acids that are useful for the design of this kind of surfaces. For instance, the amino acid lysine presents this behavior owing to the –NH_3_^+^/COO^−^ pairs and has been grafted to MSNs [181] and silica-based mesoporous bioactive glasses [182], leading to reduced bacterial adhesion and biofilm formation. A similar approach consists in using the amino acid cysteine to obtain neutral surfaces, yielding MSNs with high stability in human serum [183].

### 4.3. Addressing Bone Infections with Mesoporous Silica Nanoparticles

Besides preventing biofilm formation, the elimination of the infection is still necessary. In this sense, it is possible to engineer multifunctional mesoporous silica nanomatrices able to prevent bacterial adhesion and biofilm formation and to release antimicrobials in a controlled manner only in infected bone tissues [184,185]. In addition, there are examples of stimuli-responsive mesoporous bioactive silica-based nanomatrices able to trigger the release only in the presence of proteolytic enzymes characteristics of infected bone tissue scenarios [152,186].

In an effort to increase the efficiency of the delivery and, consequently, a reduction of the dose, the research efforts have been headed towards the development of bacteria-targeted MSNs. In this sense, the presence of positive charges on the surface of the particles increases their affinity to the negatively charged biofilm and bacteria wall. In this manner, it is easier for the particles to diffuse into the biofilm to then interact with bacteria and exert their therapeutic effect. Examples of this approach include the use of short positively charged alkoxysilanes [187] or third-generation dendrimers, with a great number of positive charges that allow permeating the bacteria wall and inducing MSN internalization [188]. Besides using positively charged MSNs, lectins have been shown to be effective in targeting and promoting internalization of MSNs into the biofilm, as a consequence of the presence of glycan-type polysaccharides in this protective matrix. In fact, the lectin concanavalin A is able to trigger this internalization and exert antibacterial effect by itself, which is even more emphasized when loading an antibiotic in the mesopores [189].

A smart approximation to enhance the possibilities that mesoporous silica materials may offer against bone infection is the incorporation of the particles within scaffolds. In the context of bone diseases, scaffolds are materials that are intended to mimic bone tissue and contribute to its regeneration. The advantages over using bare scaffolds are increased antibiotic loading capacity or controlled drug release, among others [190]. Examples of this approximation are the incorporation of silica-based mesoporous glasses in PLGA (poly-(L-lactic-co-glycolic acid)) [191] or MSNs in porous collagen gelatin [192] for the controlled release of vancomycin against bone infection. In addition, MSNs-loaded scaffolds allow the co-delivery of therapeutic compounds. In this sense, it is possible to load cephalexin within the mesopores and vascular endothelial growth factors in the scaffold structure to achieve bacteria elimination and bone reconstruction [193].

A summary of different materials for the treatment of bone infection can be found in Table 2.

## 5. Mesoporous Silica Nanoparticles for the Treatment of Osteoporosis

### 5.1. General Concepts on Osteoporosis

Osteoporosis is the most frequent metabolic disease affecting bone tissue. It is characterized by reduced bone mass and microarchitectural deterioration and results in more than 9 million fractures annually worldwide (one osteoporotic fracture every 3 s) [194], with special incidence in aged women [195]. Its origin relies on the alteration of the bone remodeling process, which consists in the removal of old bone (osteoclast) to then create new one (osteoblasts). The imbalance of this process leads to reduced bone mass and, consequently, osteoporosis.

Current osteoporosis treatments, which are not fully satisfactory, are limited to anti-resorptive drugs and anabolic agents [196,197]. Anti-resorptive drugs decrease the excess of bone resorption by targeting osteoclast activity. Examples of these compounds include bisphosphonates [198], raloxifene [199] or denosumab [200]. The excess of bone resorption can be counteracted using anabolic agents, which are compounds able to stimulate bone formation. Examples of these drugs are human parathyroid hormone [201], growth factors or siRNA [202].

Unfortunately, current treatments present some drawbacks. For instance, bisphosphonates are known to induce gastric side effects or fractures after long use. Raloxifene may cause venous thromboembolism. Moreover, cases of hypocalcemia, anaphylaxis or atrial fibrillation have been associated to denosumab. In addition, anabolic agents, such as siRNA, might be easily degraded by the harsh environment present in the organism [201]. These issues might be addressed by delivering the antiosteoporotic agents specifically to the diseased bone tissues and, consequently, the use of nanoparticles seems highly appealing.

### 5.2. Addressing Osteoporosis with Mesoporous Silica Nanoparticles

The first example of mesoporous silica materials applied for the controlled release of anti-resorptive molecules was reported by our group back in 2006, when MCM-41 and SBA-15 materials were employed for the loading and controlled release of alendronate [203]. In this sense, the introduction of phosphorous groups in SBA-15 mesoporous silica nanomatrices enhanced the loading of alendronate and induced the formation of apatite, a component of bone, making these materials promising candidates for the treatment of osteoporosis [204]. Additional examples of anti-resorptive molecules loaded in mesoporous silica-based nanoparticles are ipriflavone [205], salmon calcitonin [206] or zolendronic acid [207], with all of them showing promising results in terms of anti-osteoclast activity and osteogenesis.

A great feature of MSNs is that they allow the loading of hydrophobic compounds, consequently enhancing their bioavailability. In this sense, they allow the incorporation within their mesopores of sparingly soluble anabolic agents able to induce bone formation. Examples are the loading of dexamethasone, which induces bone regeneration through the stimulation of bone mesenchymal stems cells [208], or estradiol, which enhances the biological functions of osteoblast and inhibits the proliferation of osteoclasts [209].

Osteostain, a C-terminal peptide from a parathyroid hormone-related protein, induces strong bone anabolism through a great stimulation of osteoblastogenesis [210]. It has been shown that osteostatin-loaded SBA-15 greatly stimulate osteoblastic growth in vitro [211]. Furthermore, these osteostatin-loaded mesoporous materials have been proved to be effective in regenerating bone defects in vivo [201,212]. In addition to osteostatin, the bone morphogenic protein-2 (BMP-2), is considered to be one of the most effective growth factors to induce osteoblast differentiation and boost bone regeneration. In this sense, MSNs are useful for the co-delivery of dexamethasone and BMP-2 to achieve great bone regeneration in vivo [213]. Moreover, the residues 73–92 of BMP-2 not only promote osteogenesis and bone regeneration but also increase the internalization of bone mesenchymal stem cells of MSNs decorated with this peptidic fragment. [214].

**Figure 7:**
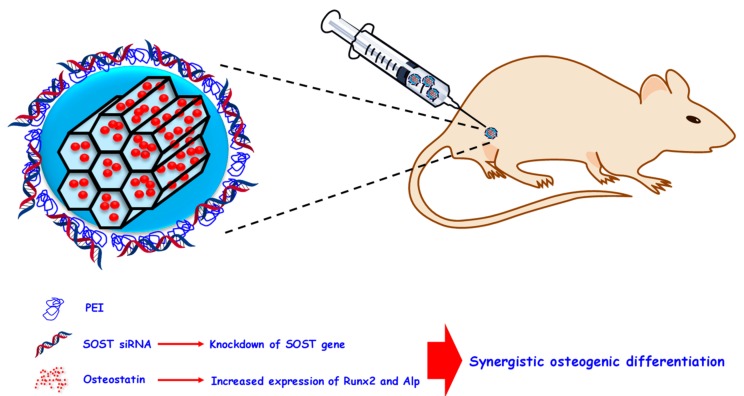
PEI-coated MSNs as anti-osteoporotic nanocarrier. Osteostatin was loaded in the mesopores and a siRNA able to knockdown the *SOST* gene was introduced within the polymeric mesh. The co-delivery of both therapeutic agents resulted in synergistic osteogenesis in ovariectomized mice. PEI: polyethyleneimine.

Aside from being useful for bone cancer treatment, siRNA molecules also find application in the treatment of osteoporosis. In this sense, the localized release of siRNA able to knockdown RANK from silica-based mesoporous bioactive glasses has been to shown to be highly effective in suppressing osteoclastogenesis and, consequently, osteoporosis [215]. A similar approach against osteoporosis was recently reported by our group using an in vivo model of ovariectomized mice (Figure 7).

MSNs can load therapeutic compounds not only in their mesopores but also within polymeric coatings through electrostatic interaction. In this sense, Figure 5 shows MSNs carrying the anabolic agent osteostatin in the pores and a specific siRNA able to knockdown the *SOST* gene interacting with a PEI coating. This gene encodes the protein sclerostin, which can inhibit the Wnt/β-catenin pathway, a major signaling carrier that regulates bone development and remodeling. Based on this, the siRNA and osteostatin-loaded nanoparticles were administered to osteoporotic ovariectomized mice, showing synergistic effects on all the bone regeneration biomarkers studied [216].

There are some metal ion species known to induce osteogenesis. For instance, copper ions enhance bone density by inhibiting bone resorption, and their incorporation in mesoporous silica nanospheres has been proved to be effective in stimulating the differentiation of bone mesenchymal stem cells into the osteogenic lineage [217]. Moreover, impregnating silica-based mesoporous bioactive glasses with Ga(III) leads to the formation of apatite together with the disruption of osteoclastogenesis and early differentiation of pre-osteoblast towards osteoblastic phenotype [218]. In addition, the osteogenic ability of Zn^2+^ ions is enhanced when the ions are co-delivered with osteostatin from silica-based mesoporous bioactive glasses [219]. Furthermore, there are nanoparticles able to stimulate bone regeneration *per se*. Examples of these kind of behavior are Au nanoparticles supported on MSNs that increase the osteogenic capability of preosteoblastic cells [220] or silica-based mesoporous bioactive glasses that are capable of reducing the bone-resorbing capability of osteoclasts [221].

A summary of all the above-described materials for the treatment of osteoporosis can be found in Table 3.

## 6. Conclusions

Bone diseases, such as bone cancer, bone infection and osteoporosis, constitute a major issue for modern societies as a consequence of their progressive ageing. Most of the current treatments present several drawbacks, leading to the deterioration of patient health and the subsequent socioeconomic impact. In this sense, the use of nanoparticles, in particular mesoporous silica-based nanoparticles, has emerged as a powerful approximation to reduce the different side effects. This type of nanoparticle presents high loading capacities, biocompatibility and can be engineered to prevent premature drug release and address the particles to the affected tissues. The different nanosystems presented here constitute reliable approximations for the treatment of bone diseases and, consequently, current research should be headed towards the effective translation of these nanomaterials into the clinic.

## Figures and Tables

**Table 1:** Summary of the different silica-based nanocarriers applied for the treatment of bone tumors.

Cell Line	Description	Reference
Osteosarcoma		
MG-63	MSNs loaded with ammonia borate as negative computed tomography contrast agents for the diagnosis of osteosarcoma;	[150]
	Silica-based mesoporous glass nanospheres for the delivery of alendronate against osteosarcoma cells and osteoclasts;	[146]
	Silica-based mesoporous glasses with osteogenic properties for the release of alendronate against osteosarcoma cells;	[145]
	Eu-doped silica-based mesoporous glass nanospheres with osteogenic properties for the release of doxorubicin;	[144]
	Influence of the different functionalizations of MSNs on their uptake by osteosarcoma cells;	[141]
KHOS	Poly-l-lysine-coated MSNs for the delivery of siRNA to knockdown polo-like kinase 1;	[159]
	MSNs with large mesopores for the delivery of siRNA to knockdown polo-like kinase 1;	[158]
	Co-loading of topotecan and siRNA to knockdown polo-like kinase 1 in dendrimer-like MSNs;	[160]
	PEI-coated MSNs for the delivery of siRNA to knockdown polo-like kinase 1;	[161]
HOS	Stimuli-responsive silica-based mesoporous glasses responsive to alkaline phosphatase overexpressed in bone tumors;	[152]
	Dendrimer-coated MSNs for the delivery of non-viral oligonucleotides;	[154]
	MSNs functionalized with singlet oxygen-sensitive porphyrin caps for release of topotecan;	[73]
	MSNs engineered for ultrasound-induced cellular uptake through the detachment of a shielding PEG layer;	[143]
	Concanavalin A-targeted and pH-responsive MSNs for the delivery of doxorubicin;	[142]
HTB-85	Silica-based mesoporous glass nanospheres with osteogenic properties for the release of doxorubicin;	[147]
U2Os	Folic acid-targeted MSNs for enzyme-responsive release of camptothecin;	[126]
UMR-106	RGD-targeted and Bi-doped MSNs for chemo-photothermal therapy and imaging;	[137]
**Fibrosarcoma**		
L-929	Ultrasound, pH and magnetically-responsive on-off gated MSNs for the delivery of doxorubicin;	[78]
	Gd-doped MSNs for magnetic resonance imagining of fibrosarcoma;	[149]
	pH-responsive MSNs for the intracellular delivery of proteins;	[153]
	pH-responsive MSNs for combined chemo-immunotherapy;	[151]
HT-1080	Influence of MSNs size on the doxorubicin release and the uptake of the particles by fibrosarcoma cells;	[148]
	MSNs decorated through an ultraviolet light-responsive linker with transferrin acting as gatekeeper and targeting agent;	[124]
	RGD-targeted MSNs for multimodal treatment of fibrosarcoma in a chicken embryo model;	[138]

**Table 2:** Summary of mesoporous silica-based materials against bone infection.

Bacteria	Description	Reference
*Escherichia coli*	Pronase-responsive gatekeepers for levofloxacin-loaded silica-based mesoporous glasses;	[152]
	Levofloxacin-loaded *Zwitterionic* MSNs with reduced protein adhesion;	[176]
	Lysine-coated MSNs to inhibit *E. coli* adhesion;	[181]
	Acid phosphatase-responsive gatekeepers for levofloxacin-loaded silica-based mesoporous glasses;	[186]
	Positively charge MSNs target the bacteria wall of *E. coli*;	[187]
	Levofloxacin-loaded MSNs coated with polycationic dendrimers destroys biofilm and internalize in bacteria;	[188]
	Levofloxacin-loaded MSNs decorated with concanavalin A targets and internalize the biofilm;	[189]
*Staphylococcus aureus*	Levofloxacin-loaded *Zwitterionic* MSNs with reduced protein adhesion;	[176]
	Lysine-coated *zwitterionic* MSNs to inhibit *S. aureus* adhesion and *S. aureus* biofilm formation;	[181]
	Lysine-coated *zwitterionic* silica-based mesoporous glasses to prevent *S. aureus* adhesion;	[182]
	Levofloxacin-loaded and positively charged MSNs targets and destroy *S. aureus* biofilm and bacteria;	[187]
	MSNs-loaded scaffolds for the co-delivery of cephalexin and vascular endothelial growth factor;	[193]
	Vancomycin-loaded silica-based mesoporous glasses contained in PLGA scaffolds;	[191]
	Vancomycin-loaded MSNs contained in collagen gelatin scaffolds;	[192]

**Table 3:** Summary of silica-based mesoporous materials for the treatment of osteoporosis.

Therapeutic Agent	Description	Reference
Anti-Resorptive Treatment		
Alendronate	First example of controlled release of bisphosphonates from mesoporous silica materials (MCM-41 and SBA-15);	[203]
	Phosphorus-containing SBA-15 mesoporous silica materials for bone regeneration and release of alendronate;	[204]
Ipriflavone	Silica-based mesoporous nanospheres for the release of ipriflavone without affecting osteoblast viability;	[205]
Zolendronic acid	Zolendronic acid-loaded MSNs/hydroxyapatite coatings on implants with enhanced inhibition of osteoclasts activity;	[207]
Salmon calcitonin	MSNs for the release of salmon calcitonin with significant therapeutic effects in vivo;	[206]
siRNA (RANK)	Silica-based mesoporous glass nanospheres to deliver of siRNA to knockdown RANK and inhibit osteoclastogenesis;	[215]
Ions	Mesoporous silica-based nanospheres for the delivery of Cu ions able to inhibit osteoclastogenesis;	[217]
	Silica-based mesoporous glasses for the release of Ga ions able to disturb osteoclastogenesis;	[218]
Particle	Silica-based mesoporous glasses reduce the bone-resorbing capability of osteoclasts *per se*;	[221]
	Au nanoparticles supported on MSNs increases the osteogenic capability of pre-osteoblastic cells;	[220]
**Anabolic Treatment**		
Dexamethasone	Alendronate-targeted MSNs for the delivery of dexamethasone to bone tissue;	[208]
Estradiol	Multilayered-coated MSNs for the delivery of estradiol from titanium substrates;	[209]
Osteostatin	Osteostatin-loaded SBA-15 mesoporous silica materials stimulate the growth and differentiation of osteoblasts;	[211]
	Osteostatin-loaded SBA-15 mesoporous materials regenerate bone in a rabbit femur cavity defect;	[201]
	Osteostatin-loaded SBA-15 mesoporous silica materials increase the early repair response in bone after local injury;	[212]
BMP-2 and dexamethasone	pH-responsive co-delivery of dexamethasone and BMP-2 protein for synergistic osteogenic effect;	[213]
	BMP-2 derived peptide-decorated MSNs for enhanced uptake in bone mesenchymal stem cells and synergistic effect of the peptidic fragment and dexamethasone;	[214]
Osteostatin and siRNA (SOST)	Enhanced osteogenic expression through MSNs co-delivering osteostatin and siRNA able to knockdown the *SOST* gene;	[216]
Zn ions and osteostatin	Co-delivery of osteogenic Zn ions and osteostatin from mesoporous silica-based glasses induces high osteogenic response;	[219]
